# Understanding frustration triggers and emotional responses in driving situations

**DOI:** 10.1038/s41598-024-76792-1

**Published:** 2024-11-19

**Authors:** Hannaneh Yazdi, Casper Wickman, Mikael Ljung Aust, Ida Selbing, Leo Kowalski, John Axelsson

**Affiliations:** 1 Department of Clinical Neuroscience, Karolinska Institutet, Stockholm, Sweden; 2 Volvo Cars, Gothenburg, Sweden; 3 Department of Learning, Informatics, Management and Ethics, Karolinska Institutet, Stockholm, Sweden; 4 Department of Psychology, Stockholm University, Stockholm, Sweden

**Keywords:** Frustration, Emotion, Behavior, Psychology, Human behaviour

## Abstract

Frustration is a complex emotional phenomenon subject to various triggers and manifested through multifaceted behavioral and affective responses. This study investigates the relationship between distinct frustration-inducing situations encountered during driving and the corresponding affective responses, focusing on the mediating role of behavioral dimensions. A total of 2244 participants answered a questionnaire on driving behavior, the likelihood of experiencing frustration in various driving situations, and affective responses in frustrating situations. Latent factors and triggers of frustration were determined using Exploratory Factor Analysis, and their relationship to driving behavior was assessed using Multiple Regression Analysis. We identified four primary clusters of situations that could trigger frustration in drivers: *Unpredictable Experiences*,* Achievement Obstacles*,* Distress Elicitors and External Distractions*. The emotions accompanying driver frustration clustered into four distinct dimensions: *Irritation*,* Anxiety*,* Boredom*, and *Embarrassment*. While anxiety-related emotions were central in all frustrating responses (i.e., all four clusters), the other emotion dimensions were specifically related to some of the frustration clusters. Additionally, different types of frustrating situations were characterized by different emotional responses. Furthermore, having more *lapses* was related to heightened frustration levels. Unraveling the complexities of frustration may aid in the further further development of traffic safety by attempting to eliminate frequent frustration triggers in driving situations.

## Introduction

Frustration is a complex and multifaceted universal experience^1^ inherent to the human pursuit of needs fulfillment, ranging from physiological needs, e.g., food and shelter, to psychological needs, from safety to self-actualization. Every day, we encounter challenges that obstruct our pursuit of our goals, and sometimes, these challenges trigger feelings of frustration. According to^2^, frustration is a goal-oriented emotional state that emerges when encountering obstacles while pursuing a desirable goal. More recently^3^, described frustration as the experience of underperformance, in the sense of facing blockages that lead to unmet desired goals. The experience of frustration can range from very simple things, like trying to scratch a spot on your back that you cannot quite reach, to very complex situations, like the failure of a rocket launch after years of dedicated preparation efforts. In our study, we define frustration as an emotional state that occurs when individuals face obstacles that impede their pursuit of a desired goal^2,4–7^.

While frustration can arise in many different situations and its manifestations are also diverse, there are some common underlying factors that act as stressors to arouse feelings of frustration. Previous research indicates that the level of frustration can be influenced by the motivation or desire to achieve a specific goal^2,4^. Additionally, frustration often arises when individuals perceive a lack of control stemming from either circumstances or the actions of others^8^. This corresponds well with personal control being recognized as a pivotal factor in well-being^9^ and indicates that individuals differ in their responses to specific frustrating situations and how they express emotional reactions depending on their level of well-being. The final behavioral response is also believed to be shaped by the emotions associated with the situation. For example, frustration that embodies feelings of anger can lead to increased aggressive behavior, whereas associations with fear can amplify the perception of risk. Thus, affective components are central to shaping the frustration response, propelling it to be suppressive, such as sadness in some situations, or more overt, like anger and irritation in other contexts^2^. Additionally, reactions can be direct responses to frustrating events where individuals are unable to express their frustration directly (e.g., by shouting or displaying aggression) due to potential consequences, leading to emotional distress and possible mental health issues, including self-harm and suicide^10^.

Individuals frequently displace their frustration onto unintentional bystanders or inanimate objects, such as slamming doors or forcefully pressing computer keyboard keys^11,12^. Previous research indicates that individuals with inadequate impulse control^13^ and disrupted attention allocation in Pediatric Bipolar Disorder^14^ are more susceptible to frustration. Daily hassles^15^, frustrations, and irritations encountered in everyday life were found to have the strongest correlations with depression, feelings of hopelessness, and thoughts of suicide among individuals with heightened emotion perception^16^. Furthermore, interpersonal factors might influence frustration, such as previous experiences with certain situations.

The World Health Organization reported that approximately 1.19 million people die each year from road traffic accidents which is the leading cause of death for children and young adults aged 5–29 years^17^. Driving represents a goal-oriented performance, starting from the moment one ignites the engine to the arrival at the destination, and while drivers normally expect a smooth ride, the driving experience can be fraught with potential frustrations, ranging from traffic jams and unruly behavior from fellow drivers to environmental challenges like slippery roads. Considering that frustration is commonly linked to goal-oriented pursuits, it seems highly likely that it also influences driving and that traffic safety could be improved if one develops an understanding of when, where, and why drivers get frustrated.

The causes of frustration in driving have been identified in various studies^18–20^, and several categories of triggers have been recognized. These include traffic-related issues (e.g., construction sites, dense traffic, red lights), in-car causes (e.g., technical defects, Human-Machine Interface issues), and external factors such as weather conditions. These findings underscore the complexity of frustration in driving, as it can stem from a wide array of sources that disrupt goal-directed actions.

The primary aim of this study is to further our understanding of the feeling of frustration in driving situations by examining whether there are distinct associations in the interplay between *frustration triggers*, affective responses, and behavioral dimensions. Specifically, we aim to assess how distinct driving-related frustration triggers are associated with affective responses (*frustration affects*) and behavioral responses (*frustration behavior*).

To achieve this aim, we first set out to identify salient frustration triggers that individuals commonly experience in driving situations. Next, we investigated which affective responses are most closely associated with experiencing frustration. Last, we assessed how drivers’ *frustration triggers* were associated with various *frustration responses* and *frustration behavior* in the driving context. The latter involves how emotional responses to frustration-inducing factors in driving relate to behavioral dimensions (violations, errors, and lapses), which are more prevalent among individuals prone to frustration.

This exploratory investigation seeks to identify overarching trends or relationships that may exist among these key elements to provide a more comprehensive understanding of frustration in the driving context. To the extent that the results of this study can help identify how and why frustration arises in driving situations, they can also support the development of strategies and technologies that help drivers cope with or mitigate frustration.

## Method

### Participants

Study participants were invited through a car company’s online community of owners, through a leasing pool, and via a car-sharing service. All data were collected using the car company’s own driver feedback platform. Participation was voluntary, and participants were promised anonymity. Instructions were provided to ensure that participants completed the questionnaires based on their personal driving experiences and perceptions, ensuring that their responses were reflective of their own interpretation. They were asked a series of questions pertaining to their driving style and about frustrating experiences while driving. A total of 2,244 (31.6% aged 45–54, M = 47.11 years) answered an online questionnaire distributed to licensed car drivers in Sweden (see Table 1 for demographics). Fourteen participants did not report their sex and age. The study was conducted according to the guidelines of the Declaration of Helsinki and was approved by the Swedish Ethical Review Authority (2020–04337). All participants voluntarily provided online informed consent before participating in the study and did not receive any compensation.

**Table 1:** Descriptive information of the participants.

	Total (N = 2244 )
Gender	
Men	1789 (79.7%)
Women	426 (19%)
Non-binary	6 (0.3%)
Preferred not to answer	9 (0.4%)
Age	
17–24	37 (1.6%)
25–34	346 (15.4%)
35–44	519 (23.1%)
45–54	709 (31.6%)
55–64	469 (20.9%)
65–74	133 (5.9%)
74+	17 (0.8%)

### Procedure and measures

Data was collected using a self-administered online questionnaire with questions about behavior and motives that could influence driving, and attitudes contributing to risky driving^21^. The survey consisted of three sections.

### Frustration situations

Frustration occurs when goal-directed actions are hindered^6^ and can be influenced, e.g., by motivation or desire to achieve a specific goal^2,4^, a lack of control^8^, and it is heightened under time pressure^22^. To capture this, we incorporated a mix of components that cause frustration, such as obstacles to goal-directed behavior and time pressure in the process of developing the frustration situations items in our study. This approach is grounded in research on frustration and previous studies on driving-related frustration, which indicate that such a combination typically resonates with a sense of frustration in participants^6,22,23^.Therefore, the list of frustration scenarios was developed based on literature reviews^18,20^; internal company reports it related to difficulties in-vehicle use, consultations with company experts (i.e., road safety, user experience, and customer satisfaction), as well as input from effective scientists. The *frustration situations* encompass traffic conditions, weather, driver mood, conditions inside and outside the car, and car systems. The participants assessed the likelihood of different frustration situations (Table 2). They were asked to “for each of the driving situations, indicate how likely it is that this will contribute to your frustration”. A 7-point Likert scale was used, ranging from 1 (strongly disagree) to 7 (strongly agree), with a neutral midpoint represented by 4 (neither agree nor disagree). The specific statements corresponding to *frustration situations* are presented in Table 3. The response scale for frustration levels does not refer to the intensity of frustration itself but rather to the probability that a situation will be frustrating.

### Frustration affects

In designing the affective responses to frustration situations, or *frustration affects*, we recognized the limited research on the diverse forms of affective and behavioral responses to driving-related frustration. Frustration is often associated with aggressive behavior, typically driven by anger^2^, which is linked to the positive arousal dimension^24^. However, to capture the full spectrum of potential affective responses to frustration, we chose not to rely solely on traditional measures of negative emotions. Instead, we adopted a more comprehensive approach by integrating validated emotional models, such as the Circumplex Model of Affect^24^, and consulting with professionals from the automotive industry and affective science. As a result, we defined 15 emotional responses (*anger*,* disappointment*,* sadness*,* stress*,* anxiety*,* boredom*,* fear*,* helplessness*,* tiredness*,* stupidity*,* irritation*,* annoyance*,* embarrassment*, and *unmotivated)* as referring to *frustration affects* in our questionnaire, reflecting varying levels of emotional arousal. Participants were asked to describe “the most frustrating situation you have ever experienced while driving” and select from the list of fifteen *frustration affects* to describe their feelings during that situation. To assess the presence and intensity of emotions experienced during the described *frustrationsituations*, we chose a bipolar scale ranging from “strongly disagree” to “strongly agree.” The participants expressed their *frustration affects* on a 7-point Likert scale, ranging from 1 (strongly disagree) to 7 (strongly agree), with 4 indicating a neutral stance (neither agree nor disagree). This approach was intended to evaluate both the acknowledgment of specific *frustration affects* and the degree of participants experienced these *frustration affects*.

### Behavioral dimensions

The original version of the Driver Behavior Questionnaire (DBQ) was developed by classifying three types of aberrant behavior: violations, errors, and lapses^25^. The DBQ is one of the most frequently used tools in research on driving behavior. In a meta-analysis by^26^, DBQ was distinguished as a predictor of traffic accidents.

Violations refer to “deliberate violations from those practices believed necessary to maintain the safe operation of a potentially hazardous system,” which can be aggressive or ordinary. Aggressive violations involve overtly aggressive acts, whereas ordinary violations are deliberate deviations from safe driving without a specifically aggressive aim. Errors are defined as the failure of planned actions to achieve their intended consequences. Lapses refer to attention and memory lapses that are generally not threatening to driving safety. Violations and errors are seen as more serious because they have the potential to cause accidents^27^. In the DBQ, participants are asked to indicate how often certain traffic situations happen to them^27^, which we identified as driving behavioral situations.

The DBQ questionnaire consists of 12 items broadly categorized into Violations, Errors, and Lapses, each comprising four items (see Table 2). Participants were asked to “choose the response that best describes your driving style.” They rated the frequency of their aberrant behaviors on a 5-point Likert-type scale (never = 1, rarely = 2, sometimes = 3, often = 4, and always = 5) and how frequently they engaged in the actions outlined. The items were derived from a three-dimensional model of the DBQ, encompassing *violations*, *lapses*, and *errors* as established by previous studies^21,27,28^. Cronbach’s alpha values for the subscales of the Driver Behavior Questionnaire were 0.447 for Violations, 0.481 for Errors, and 0.655 for Lapses. These Cronbach’s alpha values were on the lower end of the acceptable range, which is typically considered to range from 0.70 to 0.95^29^.

**Table 2:** Examples of driving behavioral situations from driving Behavior Questionnaire (DBQ) and related behavioral dimensions (*violations*, *errors* and *lapses*). This table presents an overview of selected driving behavioral situation scenarios, along with their corresponding driver behavioral dimensions according to DBQ. ,each driving behavioral situation is categorized according to its specific driver behavioral dimension.

DBQ driver behavioral dimensions	DBQ driver behavioral situations
Violations	I disregard the speed limit on residential roads.
	I get involved in ‘races’ with other drivers.
	I become angered by another driver and chase them.
	I stay in a highway lane that I know will be closed until the last minute before, forcing my way into the other lane.
Errors	I miss a ‘give way’ sign and narrowly avoid colliding with traffic having right of way.
	I attempt to pass someone that I had noticed to be signaling a left/right turn.
	I underestimate the speed of an incoming vehicle when overtaking/attempting to pass them.
	I fail to notice that pedestrians are crossing when turning into a side street from a main road.
Lapses	Intending to drive to destination A, I ‘wake up’ finding myself in destination B because the latter is my more usual destination.
	I realize that I have no clear recollection of the road along which I have been traveling.
	I switch on one thing, such as headlights, when I meant to switch on something else, such as wipers.
	I forgot where I parked my car.

### Statistical analysis

All statistical analyses were conducted using *RStudio* (Version 2023.09.1 + 494) and *IBM SPSS Statistics* (version 29.0.1.0 (171)). Explanatory Factor Analysis (EFA), a multivariate statistical procedure, was used separately to analyze the association between *frustration situations* and the likelihood of experiencing frustration, as well as how different frustration scenarios are interconnected. This was followed by using the same analysis method to investigate the interconnection of *frustration affects*. EFA has become largely utilized in psychology research^30^ and is considered the preferred method for interpreting self-reporting questionnaires^31^. EFA was selected over Principal Component Analysis (PCA) as EFA operates on the common factor model, while PCA does not^32^. Also, EFA aims to identify latent factors that best explain the observed variance in the common factor model^33^. Because this study aimed to understand the factors related to frustration triggers and frustration behaviors, EFA provides a suitable approach. The correlation matrix, the Kaiser-Meyer-Olkin measure, and communalities were examined to assess the suitability of the applied EFA for the current data. The correlation matrix was used to determine the relationships between variables in the dataset, while communalities were evaluated separately to understand the proportion of variance in each variable explained by the extracted factors in EFA. The eigenvalue values and scree plot were employed to decide which factors to retain. Factors with an eigenvalue above 1.0 were to be included. The pattern matrix, indicating the rotated solution, was presented in the results section. Promax with Kaiser Normalization as an oblique rotation was selected to run the analysis because we assume that there is no perfect independence between the variables in both frustration situations and affective responses^34,35^. Rotation converged in six iterations. Factor loadings of 0.30 or higher determined factor designation. Factors were extracted by analyzing the items loading on each factor, and they were named according to the common frustration component in grouped *frustration situations* as items in each factor and the emotion that had a higher score in the *frustration affects* loaded onto each factor. The pattern matrix, representing the rotated solution, was reported in the results. The labeling process involved assigning titles or names to factors based on the common components that were related to all *frustration situations* grouped under each factor. We finalized the EFA for *frustration situations* with a model of four factors categorized as *frustration triggers*: *Achievement Obstacles*, *Unpredictable Experiences*, *External Distractions*, and *Distress Elicitors*. The Confirmatory Factor Analysis (CFA) was conducted in the Rstudio program using the *Lavaan* package to validate the measurement model proposed in our study. The four-factor model fit indices indicated that the model fit was adequate overall. The Chi-square test statistic was significant ($\:{\:\chi\:}^{2}$(183) = 2709.943, *p* < .001). The Comparative Fit Index (CFI) and Tucker-Lewis Index (TLI) both showed values of 0.861 and 0.840, respectively, which are below the commonly accepted threshold of 0.90 but are still within a reasonable range considering the sample size. The Root Mean Square Error of Approximation (RMSEA) was 0.085, with a 90% confidence interval ranging from 0.082 to 0.087. This value indicates an acceptable fit given the context. The Standardized Root Mean Square Residual (SRMR) was 0.067, suggesting a good fit since values below 0.08 are considered acceptable. Given these results, while some fit indices suggest room for improvement, the model’s overall fit is deemed acceptable for proceeding with Multilinear Regression.

We categorized *frustration affects* into *frustration behaviors* clusters in the EFA. The resulting four-factor model, *frustration behaviors*, demonstrated the interconnectedness of *frustration affects*. We conducted preliminary analyses to ensure our data were suitable for EFA. Firstly, we examined the correlation matrix for values exceeding 0.8, finding none, indicating minimal multicollinearity. Additionally, the determinant of the correlation matrix, calculated at 0.006, exceeded the recommended threshold of 0.0001, affirming its appropriateness for EFA. Secondly, the Kaiser-Meyer-Olkin measure of sampling adequacy generated a value of 0.792 (*p* < .001), greater than the acceptable threshold of 0.5, signifying the dataset’s adequacy for EFA. Thirdly, all communalities, both initial and extraction values, fell within the range of zero to one, with no value below 0.3 except for the *disappointed* factor’s extraction value of 0.27, further supporting the appropriateness of the data for EFA. This indicates that the factor model adequately explains the variance in the data. To select the number of factors, the total initial eigenvalues for anxiety (4.135), irritation (2.282), embarrassment (1.757), and boredom (1.008) were analyzed. Based on the scree plot interpretation, we retained the first four factors, as they accounted for most of the variance in the data.

The same EFA analysis for *frustration affects* exported four *frustration behaviors*: *Anxiety*, *Boredom*, *Embarrassment*, and *Irritation*. We also used PCA here to evaluate the fit of the measurement model. The Chi-square test statistic was significant ($\:{\:\chi\:}^{2}$(71) = 1718.338, *p* < .001). The model showed the following fit indices: CFI and TLI, respectively calculated as 0.837 and 0.791, both indicating a moderate fit. RMSEA was 0.109, with a 90% confidence interval ranging from 0.104 to 0.113. SRMR was 0.089, which is below the cutoff of 0.10, indicating a good fit for this measure. In summary, the model provides a reasonable fit, and it was acceptable to proceed with the subsequent regression analysis.

After measuring the validity of the EFA by conducting CFA, we assessed the relationship between the dependent variable (*frustration triggers)* and independent variables (*frustration affects* and *behavioral dimensions*) in Multilinear Linear Regression (MLR). Regression analysis is a statistical method used to explore the relationship between a dependent variable and one or more independent variables^36^.  The EFA analysis exported factors for *frustration triggers* (*Achievement Obstacles*, *Unpredictable Experiences*, *External Distractions*, and *Distress Elicitors*) and *frustration behaviors* (*Anxiety*, *Boredom*, *Embarrassment*, and *Irritation*) that were analyzed in MLR together with behavioral dimensions (*Violation*, *Errors*, and *Lapses*).

Multilinear Linear Regression analysis generally explores the correlation between the dependent and independent variables, identifies the strength and nature of this relationship, and facilitates predictions regarding the dependent variable and the greater potential for multicollinearity among the association beta (the regression coefficient) values. Beta weights^37^ and R-squared (the coefficient of determination) are considered key indicators of MLR outcomes.

We assessed the suitability of data for Multiple Linear Regression analysis by checking multicollinearity, examining coefficient values as well as conducting residual analysis and ANOVA testing. A P-P plot was utilized to determine the normal distribution of residuals, confirming adherence to normality assumptions. Scatter plots of residuals against predicted values were inspected to ensure homoscedasticity, with residuals falling within the acceptable range of + 3 to -3. First, correlation coefficients were examined to assess multicollinearity among predictor variables. No correlations exceeded 0.70, suggesting that we do not have multicollinearity concerns in our data. Second, we ensured that the lowest coefficient value was at least one in the coefficients table. All coefficient values for predictor variables were above one, indicating moderate and tolerable influences and no multicollinearity concerns. Third, tolerance values, indicating the proportion of variance in each predictor not explained by other predictors, were assessed. Tolerance values below 0.40 might have suggested redundancy among predictors. All tolerance values in our data were above 0.40, indicating no issues with redundancy. Fourth, Cook’s distance was examined to identify influential data points that might have significantly impacted the regression model. No influential data points were found to have unduly influenced the model. Last, residuals, the differences between observed and predicted values, were checked for normality and homoscedasticity. A P-P plot was utilized to assess the normal distribution of residuals, confirming adherence to normality assumptions. Based on the examination, the data met the necessary assumptions for Multiple Regression Analysis. The data were considered suitable for regression analysis with no multicollinearity issues, acceptable coefficient values and tolerances, an absence of influential data points, and confirmation of normality and homoscedasticity of residuals. The regression coefficient (β) and associated *p*-value were used to interpret the strength and significance of the relationships.

## Results

We first aimed to address underlying factors contributing to frustration triggers and explain the interplay between affective response clusters and behavioral dimensions. Beginning with the analysis of frustration levels across twenty-one driving situations (see Table 3), we identified distinct clusters of frustration situations associated with frustration triggers by conducting Exploratory Factor Analysis (EFA). In the second step, we identified clusters that characterized the affective responses relating to frustration, termed “*affective response clusters*”. Subsequently, we employed regression modeling to predict the frustration triggers identified through EFA, incorporating both affective response clusters and behavioral dimensions. It is important to note that the measures discussed in this study, frustration situations, affective responses, and behavioral dimensions, are independent of each other.

### Identifying frustration triggers from frustrating situations

**Fig. 1:**
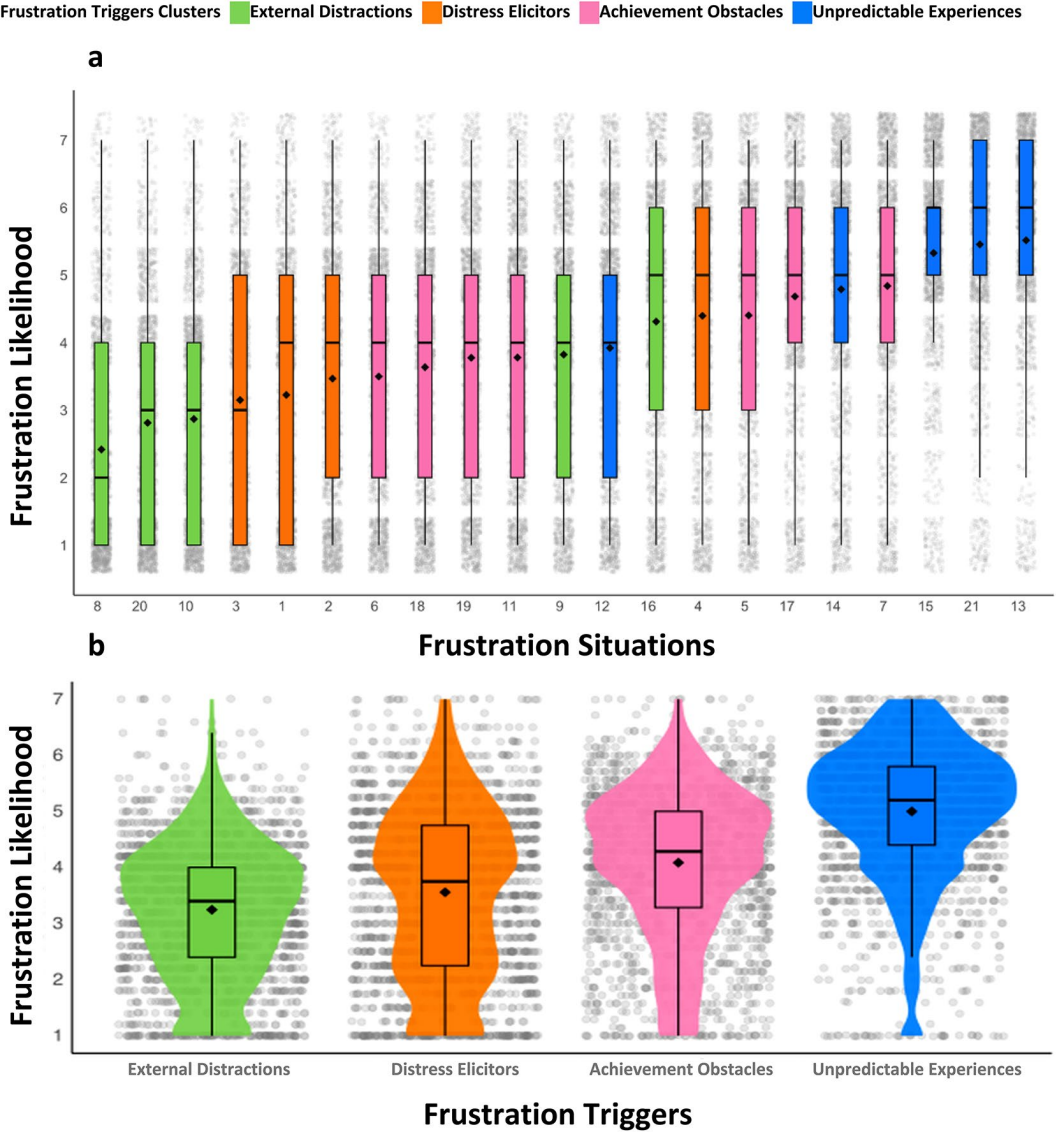
Frustration Situations and Frustration Triggers in Driving Behavior. (**a**) Mean Frustration Levels for Frustration Situations. Displays the mean frustration levels for 21 distinct *frustration situations* (see Table 3 for the items). The frustration levels are illustrated by mean values (M), standard deviation (SD), and median. Each data point is represented by a square dot (M), a box (SD), and a horizontal bold line within the box (Median), as per APA format. The grey dots represent raw data. (**b**) Correlation of Frustration Triggers with Frustration Levels through Exploratory Factor Analysis (EFA). The figure illustrates the four dimensions of factors related to frustration situations, *External Distractions*, *Distress Elicitors*, *Achievement Obstacles*, and *Unpredictable Experiences*, which represent potential sources of frustration and are extracted and plotted against the frustration levels experienced by participants. As in Figure A, M, SD, and Median are presented in the figure.

The factor analyses showed that there were four types of *frustration situations* (see Table 3; Fig. 1). These items clustered into four factors as *frustration triggers*: *Achievement Obstacles*, *Unpredictable Experiences*, *External Distractions*, and *Distress Elicitors*. The *frustration situations* with a higher likelihood of causing frustration were “when the car does not function as I expected it to,” “when the car does unpredictable things (e.g., when a system doesn’t work)” and careless driving by others (e.g., “when someone changes lanes without using the turn indicator”). *Frustration Situations* identified with higher correlation to the likelihood of frustration included *frustration situations* number 8 (“driving on a fast-moving highway”, M = 2.42, SD = 1.67, Median = 2), number 20 (described as “when the car is dirty”, M = 2.81, SD = 1.71, Median = 3) and number 10 linking to “driving when the car is full of passengers” (M = 2.87, SD = 1.64, Median = 3). The results suggest that these specific circumstances consistently elicited a higher likelihood of frustration among respondents. Eigenvalues for the retained factors were as follows: *Achievement Obstacles* (4.50), Unpredictable Experiences (2.75), External Distractions (1.90), and Distress Elicitors (1.25).

**Table 3:**
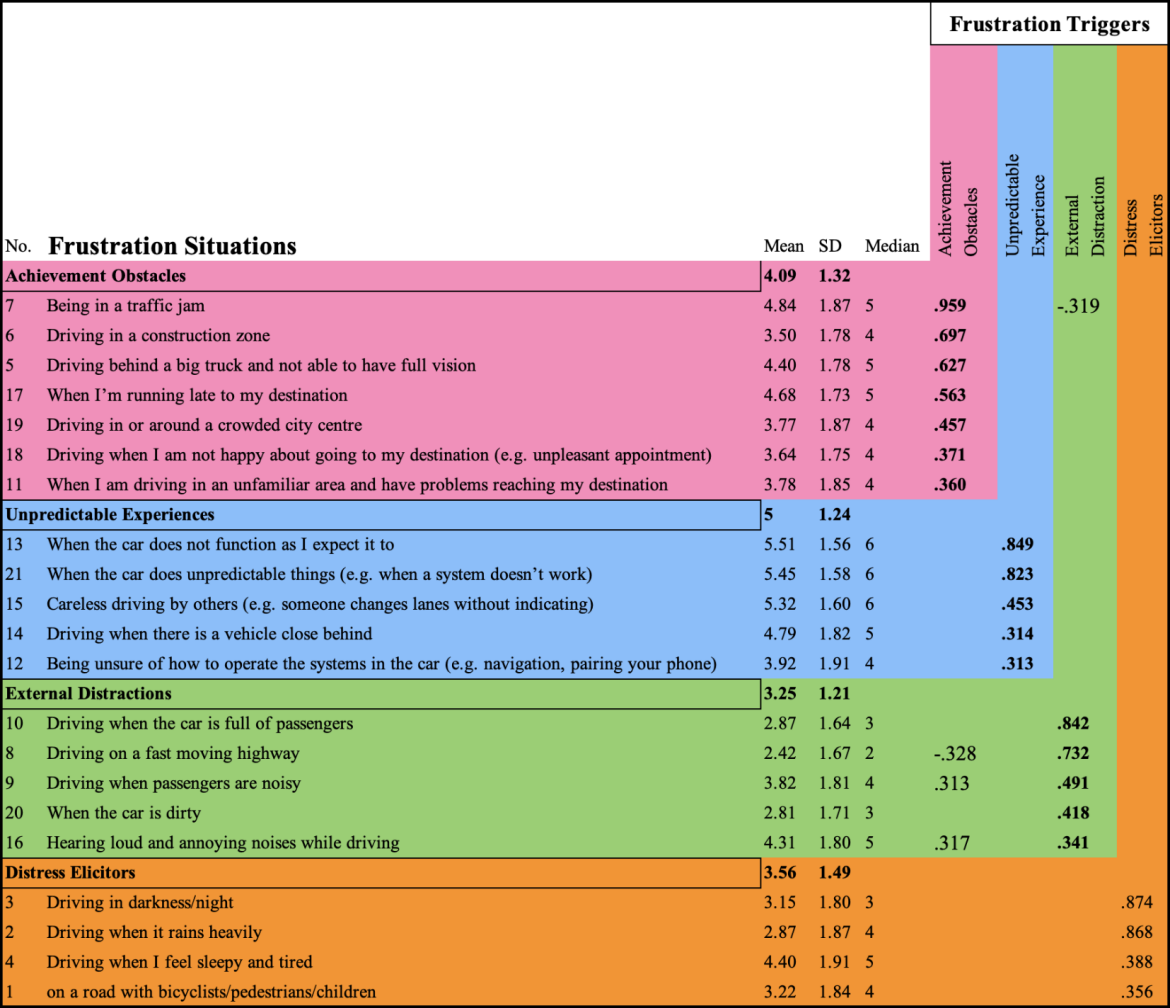
Frustration triggers extracted from frustration situations in exploratory factor analysis (EFA).The table presents EFA analysis aimed at identifying potential frustration triggers associated with frustration situations. Four presented factors were extracted by Promax with Kaiser normalization rotation method with rotation converged of 6 iterations, and values below 0.3 were not displayed. Kaiser-Meyer-Olkin measure of sampling adequacy reported 0.934. Additionally, the table includes M, SD, and median values for the frustration levels experienced in each frustration situation. Furthermore, the table specifies which frustration situations are categorized under each factor identified as frustration triggers. This comprehensive presentation allows for a detailed examination of the relationship between potential frustration triggers and frustration situations.

### Frustration affects and frustration behaviors cluster in factor analysis

**Fig. 2:**
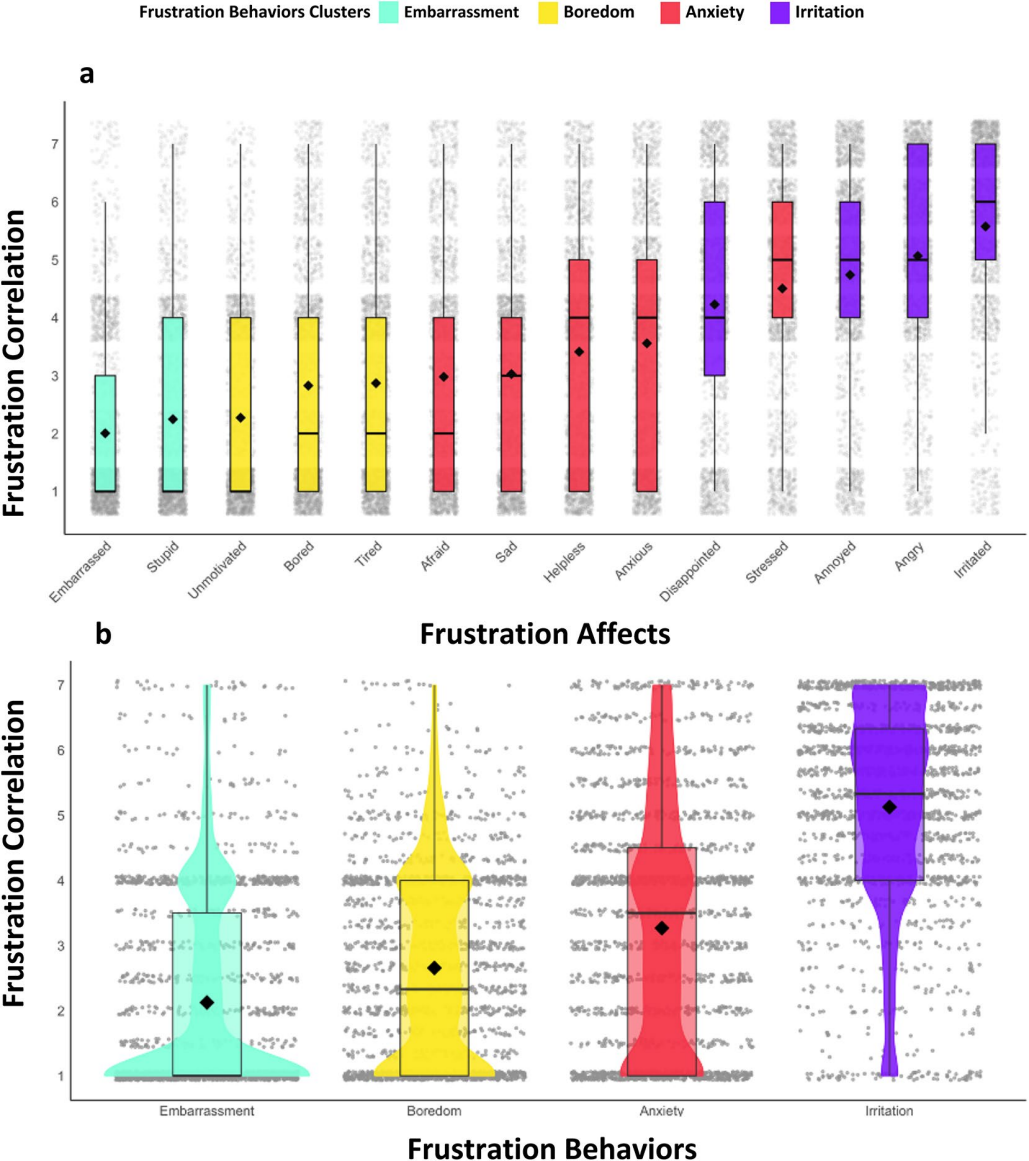
Frustration Reponses Correlated to Frustration. (**a**) Mean Frustration Correlation for Frustration Affects. The figure displays the mean frustration correlation value for 14 different frustration affects. The frustration levels are demonstrated in M, SD, and the median frustration correlation for each frustration affects. (**b**) Correlation of Frustration Behaviors with Frustration Levels through Exploratory Factor Analysis (EFA). The figure presents the results of the EFA that was conducted to identify primary factors associated with frustration behaviors. Four distinct factors, *irritation*, *anxiety*, *boredom*, and *embarrassment*, indicate potential frustration behaviors in participants’ experiencing frustration. M, SD, and median are presented in the figure.

Shifting focus to participants’ frustration affects, we delved into 14 distinct emotions in Table 4 associated with their last frustrating driving experience. The factor analysis (based on 14 items) showed that there were four *frustration behaviors* (see Table 4; Fig. 2). The *frustration affects* most strongly linked to drivers’ frustration were *irritated*, *angry*, *annoyed*, and *stressed*, while those with the weakest connection were *embarrassed*, *stupid*, and *unmotivated*.

This 4-model factors was characterized as *frustration behaviors* and named as follows: *anxiety* (*M* = 3.5, *SD* = 1.47), *irritation* (*M* = 4.9, *SD* = 1.44), *embarrassment* (*M* = 2.13, *SD* = 1.54), and *boredom* (*M* = 2.66, *SD* = 1.49). According to the EFA analysis, anxious, afraid, helpless, stressed, and sad belong to the *anxiety factor*, while irritated, angry, annoyed, and disappointed in the *irritation factor*, embarrassed and stupid belong to the factor; and lastly, bored, tired, unmotivated belong to the *boredom* factor. The *frustration affects* loaded on irritated (*M* = 5.58, *SD* = 1.69), angry (*M* = 5.1, *SD* = 1.84), annoyed (*M* = 4.74, *SD* = 1.99), and disappointed (*M* = 4.23, *SD* = 2.02) were identified as having the highest mean value among frustration affects. Lowest mean values for *frustration affects* are primarily found in the last two loaded factors in *frustration behaviors embarrassment* and *boredom*. embarrassed (*M* = 2, *SD* = 1.58), stupid (*M* = 2.25, *SD* = 1.74) in embarrassment and bored (*M* = 2.83, *SD* = 2.03), tired (*M* = 2.87, *SD* = 1.91) and unmotivated (*M* = 2.27, *SD* = 1.66) in *boredom*. Table 4 presents the factor loadings of *frustration behaviors*, communalities, and eigenvalues obtained from the factor analysis for *frustration affections*.

**Table 4:**
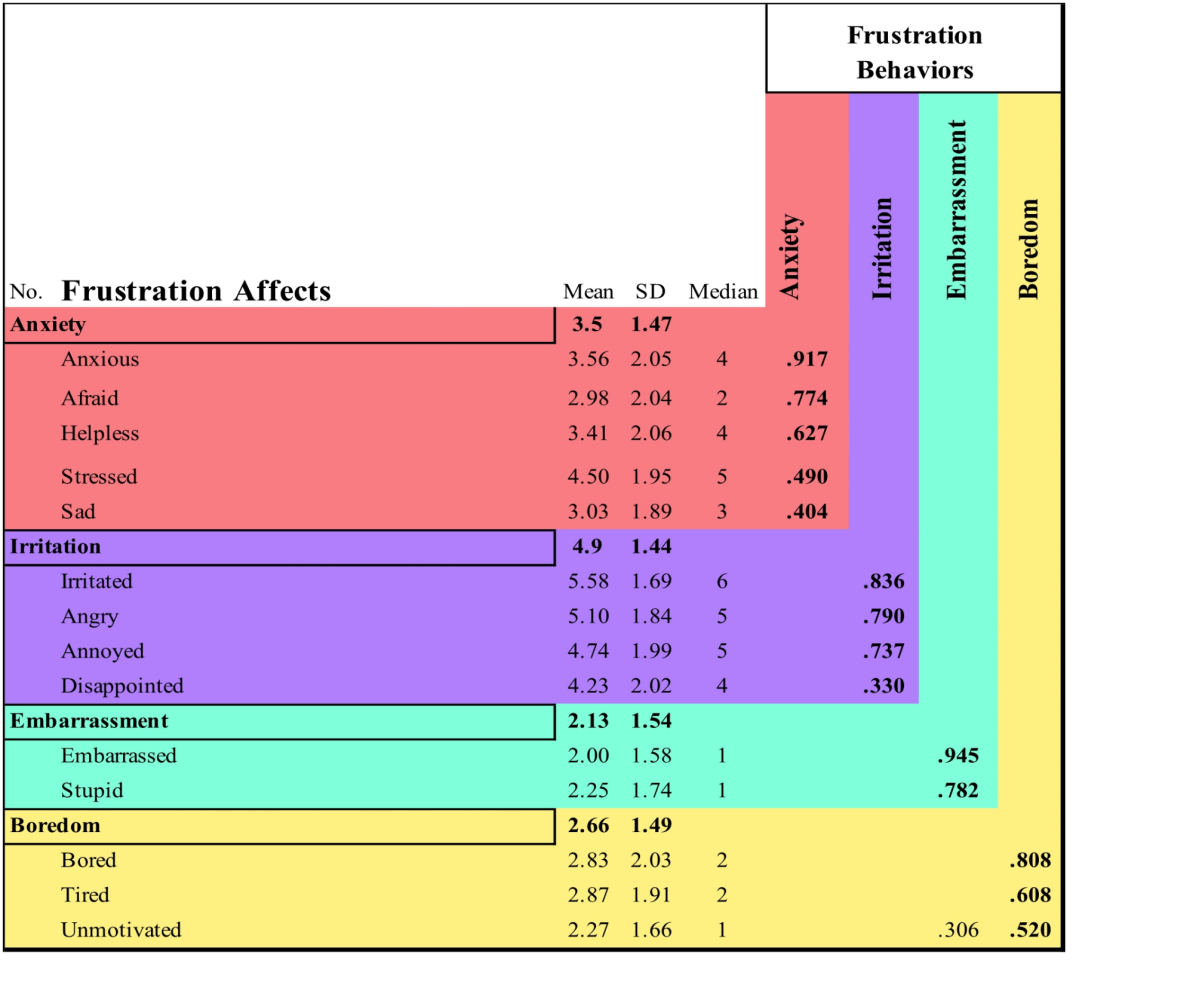
Frustration behaviors extracted from Frustration affects in exploratory factor analysis (EFA). The table presents the EFA analysis to identify potential frustration behaviors associated with frustration affects correlated to frustration. Four presented factors were extracted by Promax with Kaiser normalization rotation method with rotation converged of 6 iterations, and values below 0.3 were not displayed. Kaiser-Meyer-Olkin measure of sampling adequacy reported 0.792. Moreover, the table includes M, SD, and median values of frustration correlation experienced for each frustration affects and includes which frustration affects are categorized under each frustration behaviors cluster. This overview facilitates understanding the association between potential frustration behaviors and the experience of frustration.

### Associations Amongst Frustration triggers, frustration behaviors, and behavioral dimensions in Multilinear Regression

We used Multiple Linear Regressions (MLR) to investigate the drivers’ *behavioral dimensions* (*violations*, *lapses*, and *errors*) and *frustration behaviors* that best predict each of the four types of *frustration triggers* (see Table 5). A higher number of lapses was predictive of greater frustration, indicating that drivers with higher scores on lapses in *behavioral dimensions* were less tolerant of *frustration triggers*.

The frustration trigger of *achievement obstacles* (*R*^2^ = 0.15, *F* = 37.576, *p* < .001) showed significant association with *lapses* (β = *0.159*, p < .001) as a predictor from the behavioral dimension. This indicates that higher levels of *lapses* are associated with a higher likelihood of frustration levels in *frustration situations* involving *achievement obstacles*. Additionally, significant associations are observed for *frustration behaviors*, *anxiety* (β *= 0.142*, *p* < .001), and *irritation* (β *= 0.124*, *p* < .001), indicating that a higher likelihood of *anxiety* and *irritation* are related to a higher level of frustration in *frustration situations*. Moreover, there is a moderate association with *boredom* (β *= 0.069*, *p* < .003), suggesting a weaker but still significant relationship. However, no significant association is found with *embarrassment* (β = 0.055, *p* = .027). These results regarding *achievement obstacles* show a significant association with *lapses* in Driving Behavior Questionnaire (DBQ), indicating that individuals facing obstacles in achieving their goals are more likely to experience *lapses* and emotions such as anxiety, irritation, and, to some extent, boredom, but not embarrassment.

*The unpredictable experience situation cluster* (*R*^2^ = 0.116, *F* = 27.988, *p* < .001) was significantly associated with *violations* (β *= -0.077*,* p* < .001) and *lapses* (β *= 0.143*,* p* < .001) and feelings of *anxiety* (β *= 0.106*,* p* < .001) and *irritation* (β *= 0.205*, *p* < .001). These results demonstrated that Individuals facing *Unpredictable Experiences* also show a significant association with DBQ *lapses*. This factor correlates with *violations* and *lapses* as predictors from the behavioral dimensions , along with *anxiety* and *irritation,* similar to what was observed in *achievement obstacles*,* external distractions* (*R*^2^ = 0.102, *F* = 24.341, *p* < .001) and *distress elicitors* (*R*^2^ = 0.133, *F* = 32.623, *p* < .001) in *frustration triggers* both show significant associations with *lapses* with reported values respectively (β = 0.077, *p* < .001) and (β = 0.151, *p* < .001) as *behavioral dimensions* predictors. They also exhibit significant associations with *anxiety* (external distraction: β *= 0.139*, *p* < .001 and distress elicitors: β *= 0.18*, *p* < .001) and *embarrassment* (external distractions: β *= 0.137*, *p* < .001 and distress elicitors β *= 0.088*, *p* < .001). This indicates that higher levels of *lapses* were associated with higher frustration levels. However, DBQ *errors* did not significantly predict frustration levels, with a reported 0.495 ≤ *p* ≤ .912. Thus, while both *external distractions* and *distress elicitors* are significantly associated with DBQ *lapses* in *behavioral dimensions*, they also correlate with *anxiety* and *embarrassment*, indicating different *frustration behaviors* to compare with the first two *frustration triggers* (*achievement obstacles* and *unpredictable experiences)*,* which did not display these latter correlations*.

**Table 5:**
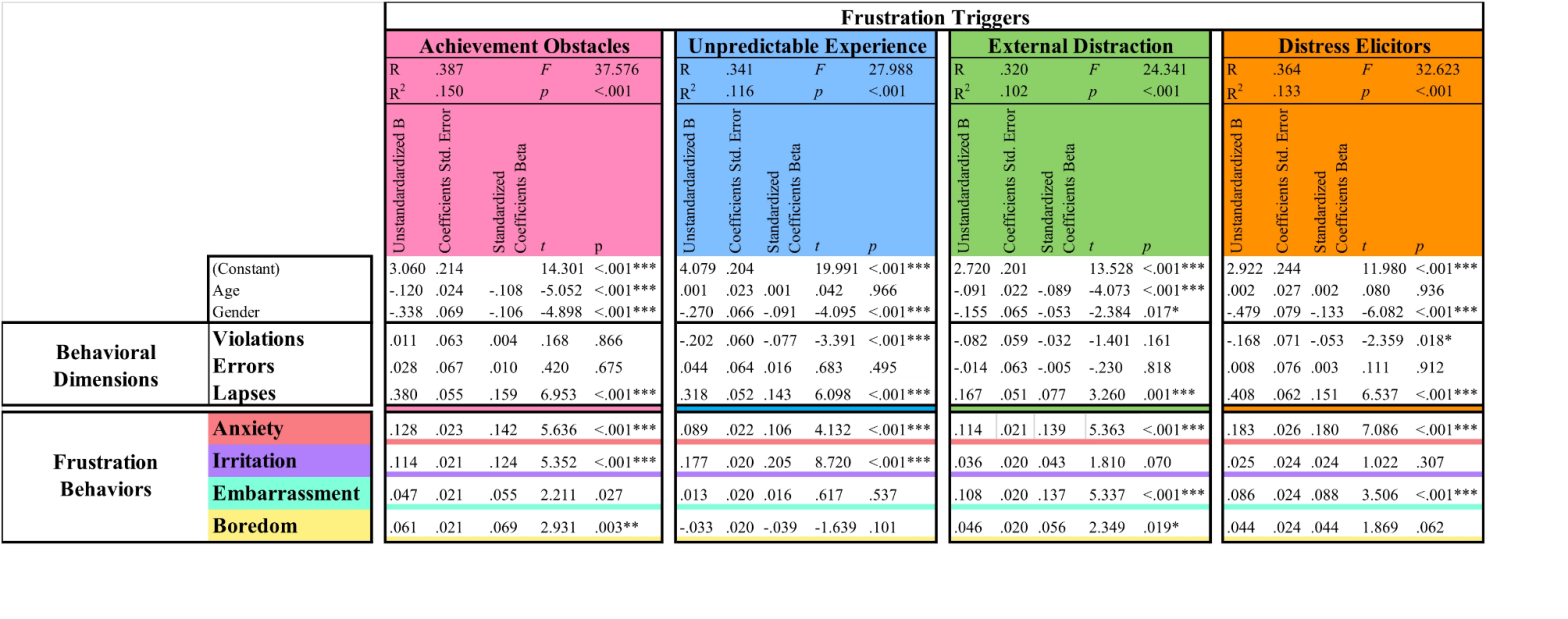
Multiple Linear Regression Analysis Predicting Frustration triggers from frustration behaviors and behavioral dimensions. The table presents the results of four multiple regression analysis models examining dependent variables: achievement obstacles, unpredictable experiences, external distractions, and distress elicitors separately associated with the predictors of frustration triggers. The predictors included in the model were age, gender, violations, errors, lapses, anxiety, irritation, embarrassment, and boredom. The overall four models were significant (*p* < .001). Standardized regression coefficients (β) and p-values are reported. P-value indicated as ***P < .001, **P < .05, *P < .01.

## Discussion

The present study examined the *frustration behaviors* and *behavioral dimensions* evoked by various *frustration triggers* within the driving context. Our findings showed that *anxiety*, *irritation*, *embarrassment*, and *boredom* were the primary *frustration behaviors* in frustrated car drivers. Moreover, the main identified *frustration triggers* in *frustration situations* could be grouped into four dimensions, *achievement obstacles*, *unpredictable experiences*,* external distractions*, and *distress elicitors*.

One objective was to characterize the factors that can trigger and shape frustration experiences in car drivers. Past research has often highlighted increased negative emotions and anger during frustrating tasks, and our results corroborate this notion. Participants primarily reported feeling irritated, angry, annoyed, and stressed when faced with *frustrating situations*. Also, our findings align closely with the updated frustration-aggression hypothesis proposed byBerkowitz^5^ who criticized the original theory posited by Dollard et al.^2^ suggesting that different frustration triggers can elicit varied affective responses. For instance, frustration caused by *achievement obstacles* and *unpredictable experiences* is here associated with heightened anxiety and irritation, whereas triggers such as *external distractions* and *distress elicitors* may lead to feelings of embarrassment, a more subdued form of frustration. Berkowitz^5^ further posited that frustration can incite aggressive tendencies only when coupled with increased negative affect. Hence, we suggest that *achievement obstacles*, *unpredictable experiences*, *external distractions*, and *distress elicitors* are the primary *frustration triggers*, heightening the likelihood of frustration among drivers.

Norman^38^ makes a distinction between *errors* and *lapses*, explaining that an error occurs when the intention is inappropriate, whereas a lapse occurs when the action does not align with the intended plan. Our results indicate that driver frustration has a strong connection to *lapses*, such as attempting to drive away from traffic lights in the wrong gear. As each aberrant of behavioral dimensions (i.e., *violations*, *errors*, and *lapses*) has different psychological origins and they demand different strategies for coping, further exploration of the *lapses* behavioral dimension exclusively in relation to the experience of frustration on the road, seems like a promising way forward. Understanding the underlying mechanisms of how *lapses* contribute to frustration is likely to aid the development of effective coping strategies that can mitigate negative emotions while driving.

In line with previous research^20^, our results emphasize that frustration can arise from various sources, including traffic-related issues, in-car factors, and self-inflicted causes. Bosch^20^ identified frustration triggers such as construction sites, dense traffic, and technical defects, along with coping strategies like distraction or seeking solutions. Our study extends these insights by categorizing frustration situations into four distinct triggers: achievement obstacles, *unpredictable experiences*, *external distractions*, and *distress elicitors*. By pinpointing specific *frustration triggers* and linking them to *behavioral dimensions* and *frustration behaviors*, our research provides a more nuanced understanding of the complexity of driver frustration. This knowledge could inform the development of frustration-aware systems, as suggested by^20^, which could offer real-time assistance based on the detected sources of frustration^39^.

Recent studies have explored various strategies to mitigate driver frustration, including providing feedback that help drivers regulate their emotional responses^40^. A key area for practical application is the role of Advanced Driver Assistance Systems (ADAS) in managing driver frustration. ADAS technologies, such as adaptive cruise control, lane-keeping assistance, and traffic jam assist, can significantly reduce the occurrence of frustrating situations by automating certain driving tasks and providing real-time feedback. These systems are particularly beneficial for drivers who are prone to lapses in behavior, such as unintentional mistakes like swerving between lanes on the highway. By minimizing the cognitive load and frustration associated with challenging driving conditions, ADAS has the potential to lessen the likelihood of frustration-induced aggressive behavior.

In the traffic psychology and behavior literature, *violations*—not *errors* or lapses—have typically been linked to “aggressive driving episodes”^41^. However, among elderly drivers, elevated error and lapse scores have been associated with active accident involvement, while passive accident involvement has been correlated with high *lapses* factor scores^42^. Interestingly, our findings demonstrate that violations do not significantly influence frustration levels in driving. Understanding why this is the case warrants further investigation.

### Limitations and recommendations

Our study used online questionnaires to collect data, yielding a substantial dataset, but since it was conducted as a cross-sectional study, causal relationships could not be determined. Furthermore, future studies would benefit from applying both a more longitudinal approach to study change over time, as well as finding a way to assess affective responses promptly after the experience rather than with a delay to minimize the temporal gap between the occurrence and the rating of emotions. As this is challenging for naturalistic studies, we propose conducting controlled studies focused on frustration to delve deeper into the triggers and real-time affective responses in driving. Additionally, the study did not measure driving experience and driving frequency, which might have influenced participants’ exposure to frustrating events and their ability to interpret and respond to such events. Moreover, because interpersonal components (i.e., impulse control, disturbed attention, and level of emotion perception) are associated with the experience of frustration, further analysis of personality traits (i.e., anger and anxiety traits^43^) and mental disorders might be necessary to understand the experience of frustration in greater depth. Also, the lower end of the acceptable range for Cronbach’s alpha may impact the reliability of the scales, particularly for the Violations and Errors dimensions. Future research should explore the implications of using theory-based measures, such as PANAS-X^44^, alongside EFA-based approaches to better understand the relationship patterns between frustration triggers and frustration behaviors, including variables like attentiveness, anxiety, sadness, and anger, which have established theoretical and practical relevance in driving frustration contexts. Furthermore, future research might benefit from employing a single-polar scale that more directly captures the intensity of frustration affects.

## Conclusion

Our study examined an interplay between *frustration triggers*,* frustration behaviors*, and driving behavioral dimensions, providing a more nuanced understanding of frustration within the driving context in a cross-sectional approach. Overall, drivers exhibiting a propensity for *lapses* are also more prone to experience frustration in driving situations. Hence, comprehending the root causes of these lapses might significantly enhance our understanding of frustration in driving and aid in developing mitigation strategies, be they vehicle technology or driver behavior-based. We found four clusters of *frustration situations* as *frustration triggers*: *external distractions*, *distress elicitors*,* achievement obstacles*, and *unpredictable experiences*. The primary *frustration behaviors* linked to driver frustration include heightened levels of *anxiety* and *irritation*, while *boredom* was very weakly related to frustration.

## Data Availability

We intend to share the data and in case to request the data from this study contact Hannaneh Yazdi.
